# High-fidelity and high-resolution phase mapping of granites via confocal Raman imaging

**DOI:** 10.1038/s41598-021-87488-1

**Published:** 2021-04-13

**Authors:** Krishna C. Polavaram, Nishant Garg

**Affiliations:** Department of Civil and Environmental Engineering, University of Illinois at Urbana-Champaign, Newmark 2129, 205 N. Mathews, Urbana, IL 61801 USA

**Keywords:** Mineralogy, Imaging techniques

## Abstract

In physical sciences such as chemistry and earth sciences, specifically for characterization of minerals in a rock, automated, objective mapping methods based on elemental analysis have replaced traditional optical petrography. However, mineral phase maps obtained from these newer approaches rely on conversion of *elemental* compositions to *mineralogical* compositions and thus cannot distinguish mineral polymorphs. Secondly, these techniques often require laborious sample preparations such as sectioning, polishing, and coating which are time-consuming. Here, we develop a new Raman imaging protocol that is capable of mapping unpolished samples with an auto-focusing Z-mapping feature that allows direct fingerprinting of different polymorphs. Specifically, we report a new methodology for generating high fidelity phase maps by exploiting characteristic peak intensity ratios which can be extended to any multi-phase, heterogenous system. Collectively, these enhancements allow us to rapidly map an unpolished granite specimen (~ 2 × 2 mm) with an exceptionally high accuracy (> 97%) and an extremely fine spatial resolution (< 0.3–2 µm).

## Introduction

Characterizing the chemical and phase composition of inorganic materials is central to advances in almost all of physical sciences, and this is also important to accelerate materials discovery^1^. In order to accurately discern the composition and distribution of chemical phases present in an inorganic material, many tools are available in the analytical toolkit. Specifically, for minerals in a rock, these include traditional optical petrography on thin sections^2^, X-ray Diffraction (XRD) on bulk specimens^3^, Electron Probe Micro Analysis (EPMA)^4,5^, automated Scanning Electron Microscope—Energy Dispersive X-ray Spectroscopy (SEM-EDXS)^6^, infrared spectroscopy (IR) (near^7^ and mid^8^), and Raman imaging^9,10^. Each of these techniques have their own set of capabilities and limitations, and due to their complementary nature, can often be combined to produce information rich datasets at multiple scales—as is being seen in the emerging field of correlative characterization^11,12^.

However, it is often not fully clear which technique is most suitable under what scenario. Specifically, all of these techniques have their own set of ideal sample requirements in terms of thickness and roughness as well as limits on spatial resolutions and surface areas that can be mapped. For e.g., for traditional optical petrography thin and polished sections are an absolute must whereas for Raman imaging bulk, rough surfaces can be mapped^13^. Finally, other practical matters such as cost (operational and capital), time (scan duration, analysis time), and ease of operation can also play an important role in selecting a particular tool. Hence, a direct comparison between these techniques is of great interest.

In this study, we report a novel high-fidelity mapping protocol with Raman imaging which is compared with the commonly used SEM-EDXS based mineral phase mapping (also known as QEMSCAN^14^). We have chosen 3 unpolished granite specimens given the abundance of silicates in Earth’s crust for this protocol. Granite is a heterogeneous mixture of many minerals such as feldspar (plagioclase and orthoclase), quartz, mica (biotite and muscovite), and amphibole, whose interlocked and intertwined crystalline grains form spatially complex microstructures. This new Raman mapping protocol exploits characteristic peak intensity ratios for each mineral and then uses sequential image processing to produce definitive and high-resolution Raman phase maps (< 0.3–2 µm) for areas of ~ 2 × 2 mm in size. Similar phase maps from SEM-EDXS on the same sample area of ~ 2 × 2 mm were also obtained using elemental analysis for comparison. By comparing the composite phase maps obtained from both elemental (EDXS) and mineralogical (Raman) approaches, we report a high level of agreement between the two techniques (> 97%) but find a distinct advantage with Raman imaging which produces an overall lower % of non-assigned region and an overall higher spatial resolution for the mapped region.

## Results and discussion

### Characteristic peak intensity ratios as valid proxies for mineral identification

**Figure 1:**
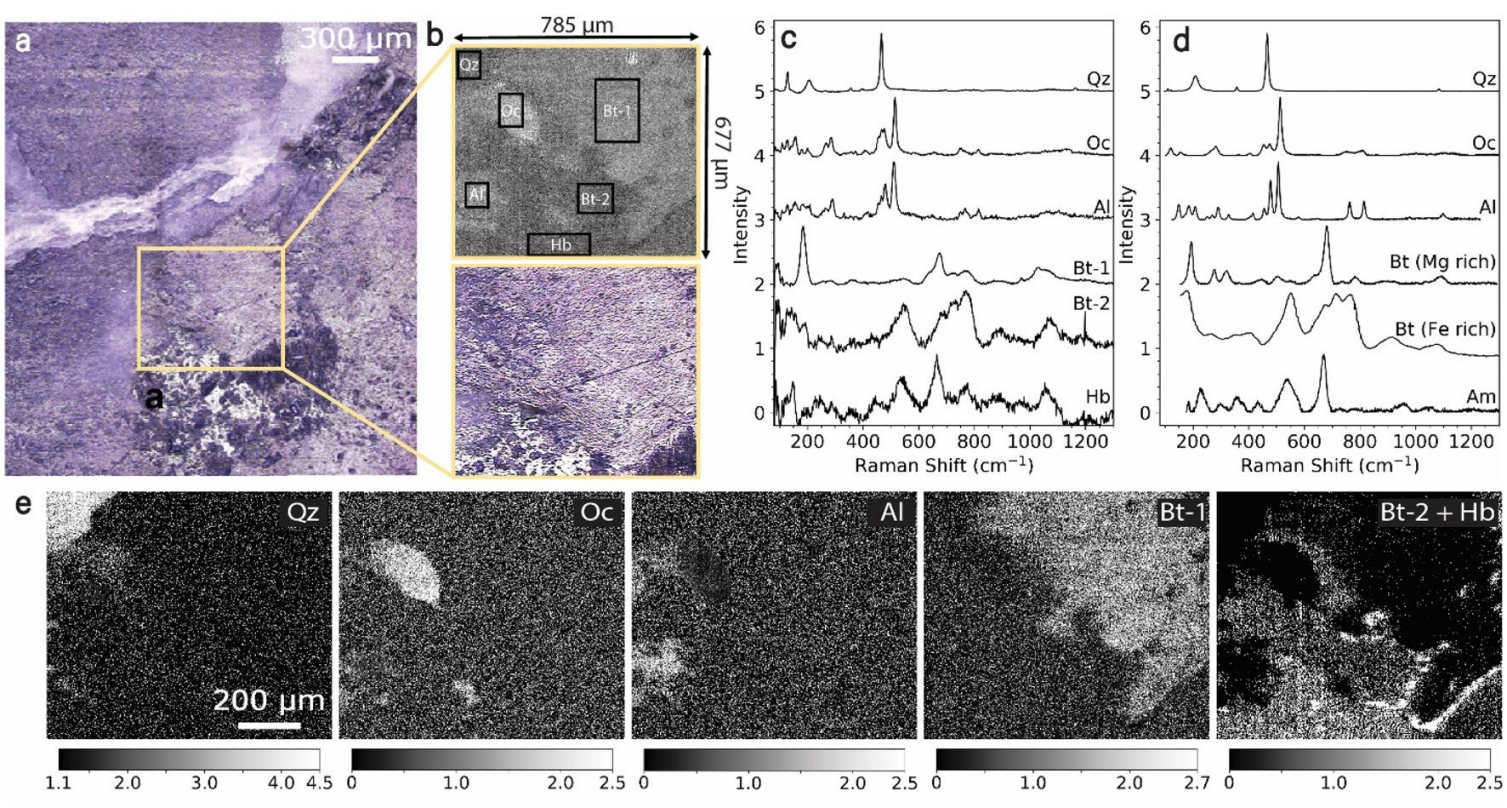
Granite-1 specimen and associated Raman spectra. (**a**) Stitched optical image from 12 tiles. (**b**) Optical tile and preliminary Raman contrast map of an individual tile at 10 × magnification. (**c**,**d**) Normalized experimental (**c**) and reference (**d**) Raman spectra for the observed minerals. Reference spectra have been obtained for Quartz (RRUFF id: R040031), Orthoclase (RRUFF id: R040055), Albite (RRUFF id: R040068), Biotite (Mg rich) (RRUFF id: R050068), Biotite (Fe rich)^21^, Amphibole (RRUFF id: R110203). (**e**) Contrast images showing presence of minerals obtained by plotting ratios of characterized peak intensities. Legend: *Qz* quartz (SiO_2_), *Oc* orthoclase (KAlSiO_3_), *Al* albite (NaAlSiO_3_), *Bt* Biotite (K(Mg,Fe)_3_(AlSi_3_O_10_) (OH)_2_), *Hb/Am* Hornblende/Amphibole ((Ca,Na)_2_(Mg,Fe,Al)_5_(Al,Si)_8_O_22_(OH)_2_.

Given the importance of silicates and their widespread availability in not only Earth’s crust^15,16^ but also other terrestrial planets such as Mars^17^ and Mercury^18^, we chose granite to develop our novel mapping protocol. In Fig. 1a, we report an optical image of the area of interest (2.21 × 2.36 mm) on granite-1 sample which was obtained by stitching together 12 individual optical images (tiles) collected with an optical microscope. From this stitched optical image, we have chosen a single optical tile of 785 µm × 677 µm that contains all the different mineral phases expected in this granite sample and reported it along with its preliminary Raman contrast map in Fig. 1b. This preliminary Raman contrast map refers to an intensity map where the sum of intensities at 185.1 and 510.9 cm^−1^ (chosen preliminarily, specific to this sample) was plotted which gave a reasonable contrast variation for illustrating different phases. Granite is expected to have the presence of silicates such as quartz, albite, orthoclase, hornblende, and biotite^19,20^ and these were found to be present in the sample as shown by the average experimental spectra (Fig. 1c) of the boxed areas in Fig. 1b. For reference, standard Raman spectra from RRUFF in Fig. 1d are also plotted for comparison with the experimental spectra in Fig. 1c, indicating a close match with assigned mineral phases for quartz, orthoclase, and albite. We note that we found two different kinds of biotite in this particular sample and these are likely to be different endmembers from the biotite series. The closest standard Raman spectra obtained for hornblende was that of its parent mineral, amphibole, which has identical characteristic peak locations. Raman spectroscopy can readily detect such different polymorphs as relative peak intensities are a direct function of the local atomic structure^21^. Selected studies in the past simply use a single peak intensity or peak area to map the presence of a given mineral^22,23^, however, that can lead to inaccurate results due to overlapping peaks in similar minerals. A previous study also used multivariate curve resolution—alternating least squares (MCR-ALS) to spatially resolve and map different phases of TiO_2_ along with quartz and epoxy^24^. However, approaches using MCR-ALS require different sets of starting parameters to be tested until satisfactory results are obtained to ensure optimum unmixing of pure components^25^ and tends to be computationally intensive. Moreover, the Raman imaging approach presented here can detect ‘overlap’ regions where multiple minerals are present. Here, in Fig. 1e, we have plotted phase maps by adopting characteristic peak intensity ratios as a way to identify and fingerprint unique minerals. For e.g., we have chosen the peaks X and Y (representing 2 stretching bands) and obtained a ratio of X/Y and used that to plot a contrast image which results in a phase map. The characteristic peaks observed in the spectra for quartz (466, 207.2, 129.6 cm^−1^), albite (510.9, 478.9 cm^-1^), orthoclase (515.2, 474.6 cm^−1^), biotite (185.1, 675.7 cm^−1^) and hornblende (665.2, 769.4 cm^−1^) are in good agreement with previous studies^21,26–28^. As shown in the scale bar of Fig. 1e, the peak ratio values for each peak set (detailed in methods) are unique and allow unambiguous mineral identification.

### Developing a Raman phase mapping protocol using a mineralogical approach

**Figure 2:**
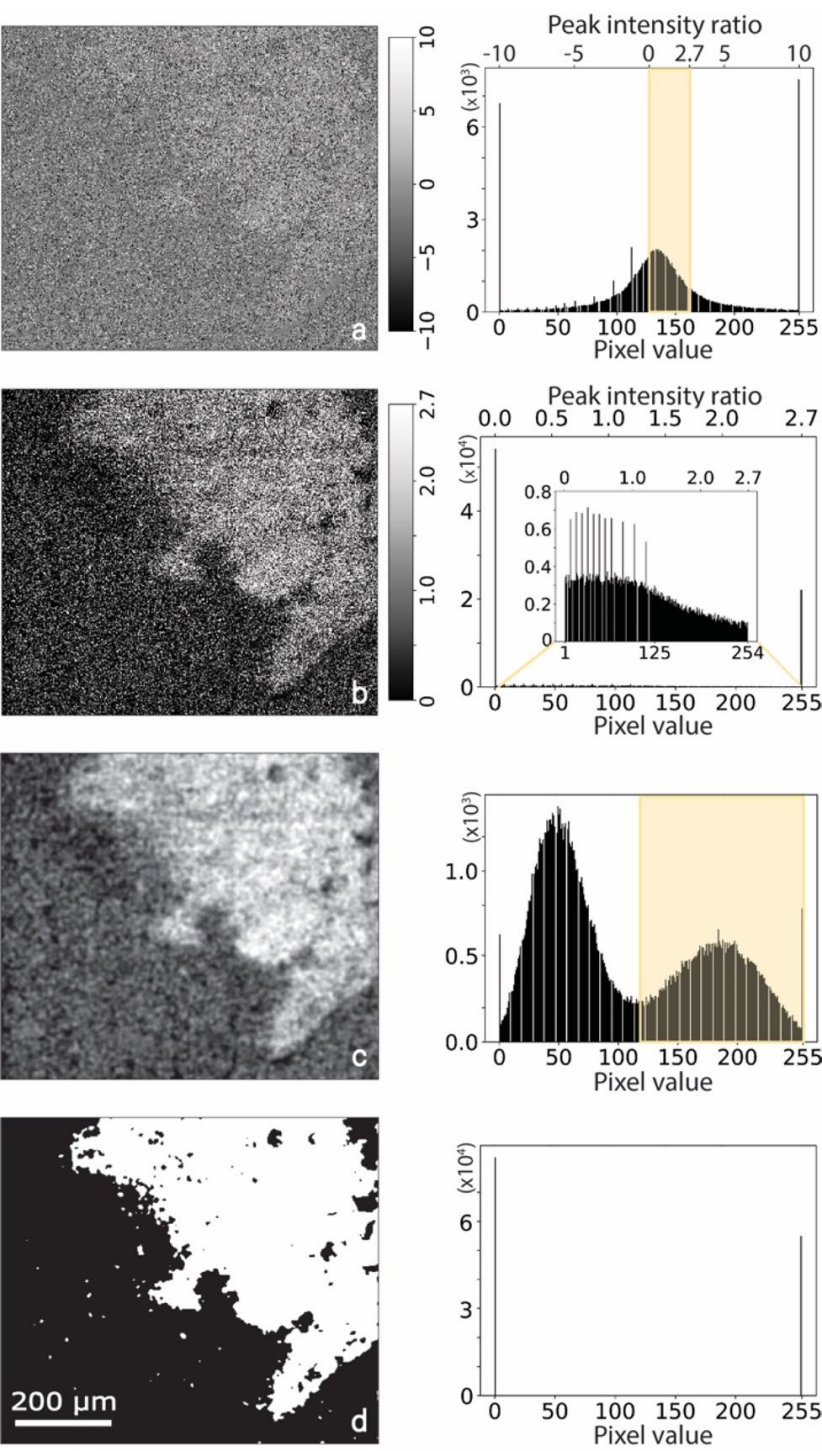
Methodology illustrating the process of obtaining definitive phase maps. (**a**) Contrast image obtained after choosing intensity ratios with ratios ranging from − 10 to + 10. (**b**) Contrast image after applying intensity ratio range from 0 to 2.7, 2.7 being characteristic to biotite. (**c**) Contrast image after applying bandpass filter corresponding to biotite peak in the histogram. (**d**) Definitive phase map of Biotite-1 in tile 8.

**Figure 3:**
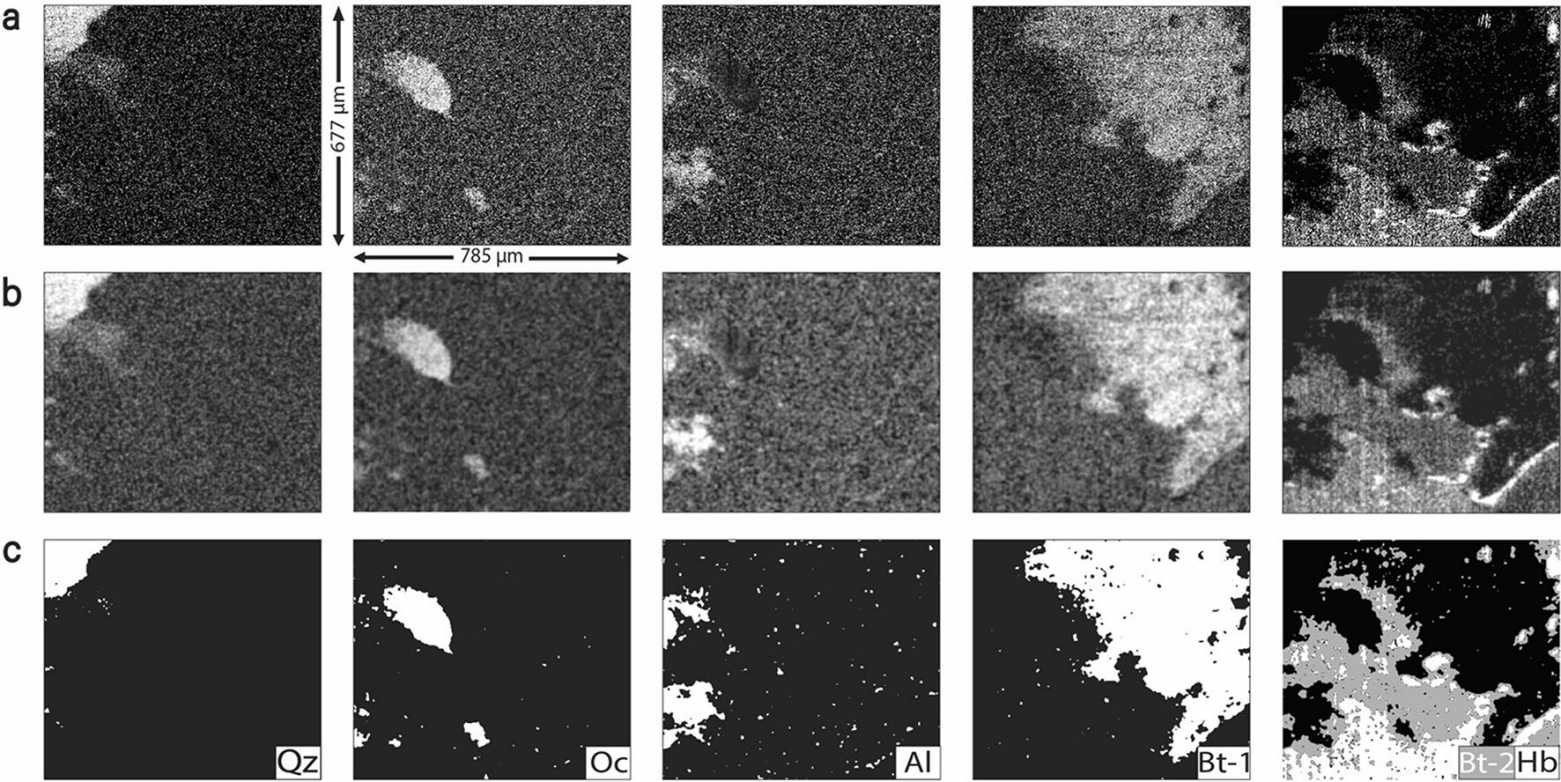
Phase maps of all minerals in the selected tile that have been obtained using the developed methodology. (**a**) Raw Raman images obtained from characteristic peak intensity ratios, (**b**) Bandpass filtered image for separating regions of interest. (**c**) Definitive phase maps of minerals. Legend- *Qz* Quartz, *Oc* Orthoclase, *Al* Albite, *Bt* Biotite, and *Hb* Hornblende.

Phase maps have spatial information about the presence and absence of a particular mineral. In the past, studies have generated phase maps by plotting contrast images using peak intensities^15,29,30^, but well-segmented and definitive images are limited. Here, we have developed a four-step methodology to obtain definitive phase maps which has been illustrated for biotite-1 mineral in the same tile which was used in Fig. 1b–e. The two most intense peaks of biotite-1 are at 185.1 cm^−1^ and 675.7 cm^−1^ which corresponds to Si–O-Si bending and translational M–O (M = Mg, Fe) respectively^31^. In order to obtain phase maps, these peaks were assigned as characteristic peaks whose peak intensity ratio had a unique value of 2.7 for this mineral. This characteristic peak intensity ratio was used to generate a contrast image (Fig. 2a) whose gray value at every location is proportional to that ratio. Figure 2b shows the image filtered for the required range of ratios (0 to 2.7) which was then subjected to a Fourier transform to segment the required region as shown in Fig. 2c. The accompanying histogram of this image is expected to have peaks corresponding to areas where the mineral is present and absent. Finally, this image in Fig. 2c was then thresholded using the Isodata^32^ algorithm in *ImageJ* resulting in a phase map which is a definitive binary image that shows the presence and absence of the mineral (Fig. 2d). This process was subsequently adopted for all minerals observed in the selected tile and the stepwise results are shown in Fig. 3. All of these minerals are common in granite^33,34^, and using this new methodology, an unambiguous identification is now possible.

### Mineral phase maps using an elemental approach

**Figure 4:**
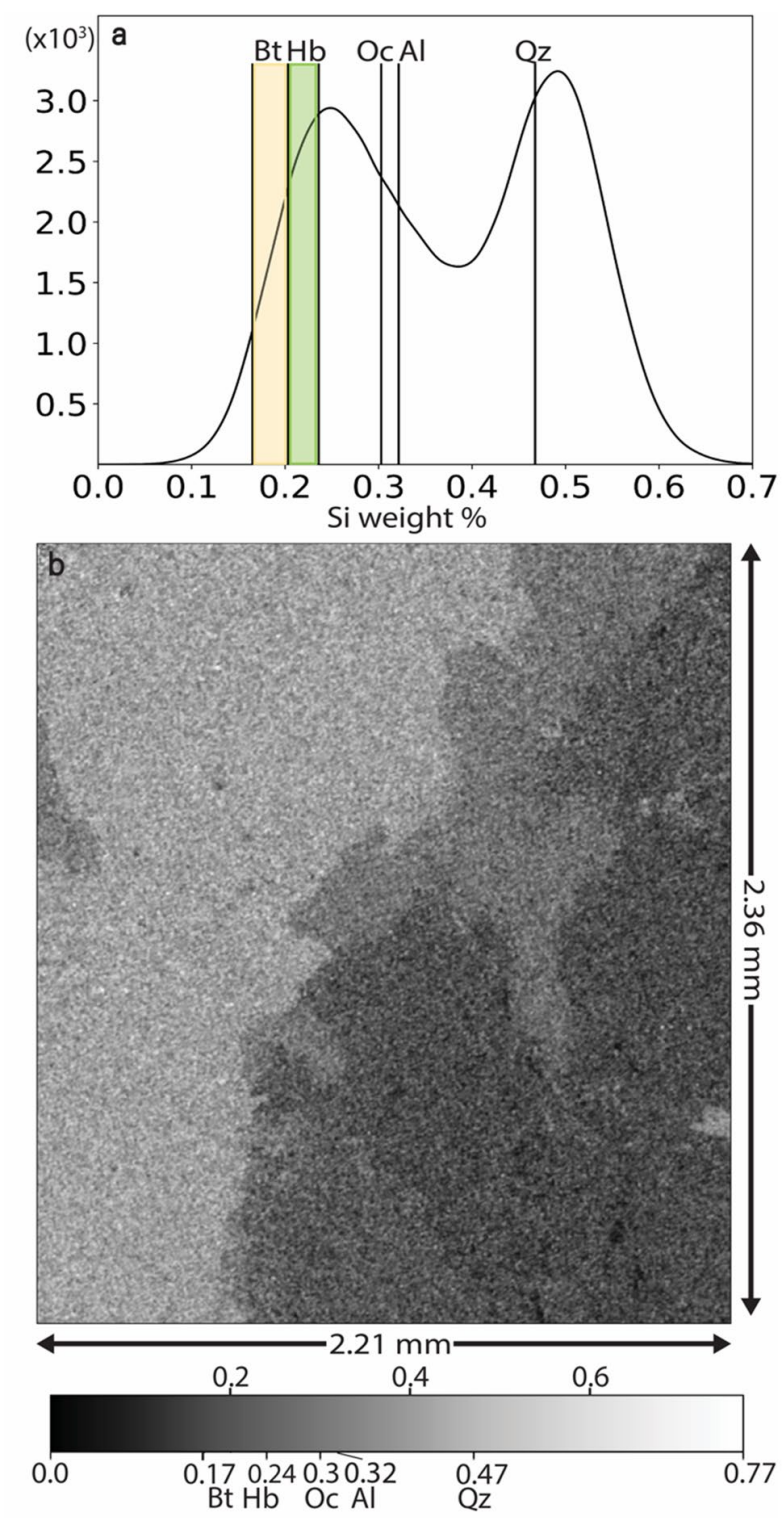
EDS elemental map analysis to differentiate mineral regions. (**a**,**b**) Si weight % histogram (**a**) and map (**b**). The Si weight % has a theoretical value of 0.3 for Orthoclase, 0.32 for Albite, and 0.47 for Quartz. The values for biotites fall in between 0.17 and 0.21 and that of hornblendes fall in between 0.21 and 0.23. The first peak in the histogram occurs as a result of overlap of multiple peaks of different minerals such as biotite, hornblende, orthoclase, and albite whereas the second peak completely corresponds to quartz which has a much higher value owing to the fact that it has high silicon content.

EDS coupled with SEM is also another tool to obtain the spatial distribution of different minerals by mapping the elements^35–37^. In general, however, it is challenging to observe and compare data collected on the same area of the sample when there are variations in sample orientation, composition, and topography. In a previous approach^38^, compositional differences were observed between different phases using combined EDS elemental maps and back scattered images for quantitative analysis of minerals. Here, however, we have employed a different approach of using the gray value of a point to map it to a mineral based on characteristic Si weight % values. Elemental maps were subjected to ZAF (Atomic number, Absorbance and Fluorescence) corrections to obtain weight % maps resulting in pixels with gray values which are proportional to the weight of the elements. Then, we generated a grayscale Si relative weight % map by combining all the elemental weight % maps using Eq. 1 and have reported the outcome in Fig. 4.

1 $${\text{Gray}}\;{\text{value}}\;{\text{of}}\;{\text{relative}}\;{\text{weight}}\% \;{\text{map}} = Wt_{Si} \cdot \left( {\mathop \sum \limits_{i} Wt_{i} } \right)^{ - 1}$$

where *Wt*_*i*_ is the weight of *i*th element (*i* = 1–8: Si, Al, Fe, K, Na, O, Ca, and Mg).

This map shows the Si relative weight concentration at every point in the scan area for which phase assignment was done using theoretical values based on known chemical formulae^39^ with a tolerance of 0.015%. For minerals such as biotite and hornblende which occur as a series of minerals in nature with known end-members^40^, the corresponding Si weight % ranges were used. The mapped minerals were color coded to generate a composite EDS map which is explained fully in the next section.

### Multi-modal correlative imaging

**Figure 5:**
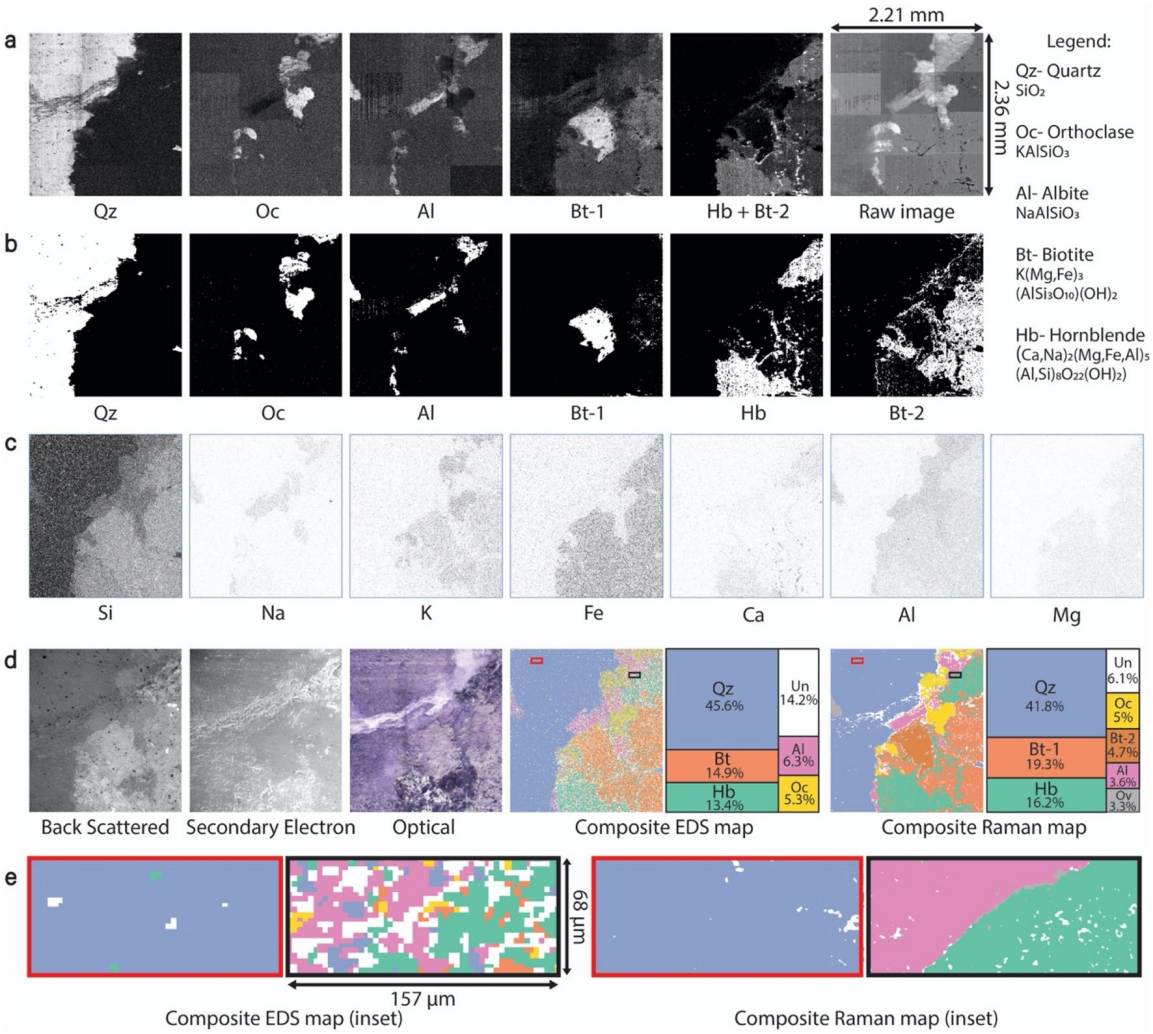
Multi-modal correlative imaging for granite-1 specimen. (**a**) Raw Raman images obtained from characteristic peak intensity ratios. (**b**) Raman phase maps obtained using the developed 4-step methodology (white reflects presence of mineral, resolution is 1.96 μm/pixel). (**c**) EDS elemental maps (dark reflects higher concentration of element, resolution is 5.12 μm/pixel). (**d**) SEM images (light reflects heavier element in back scattered image, resolution is 3.4 μm/pixel), optical image (resolution is 3.6 μm/pixel), composite EDS and Raman composite phase maps. (**e**) EDS and Raman composite phase maps from red and black boxes marked in d. (resolution of Raman map is 0.39 μm/pixel whereas that of EDS remains the same—5.12 μm/pixel); Since human perception of colors can be biased and different colors can change the visual representation, neutral color schemes for composite maps were adopted from ColorBrewer^45^.

By assimilating both the EDS and Raman imaging maps developed in previous sections along with backscattered and secondary electron imaging, we now report a multi-modal correlative dataset for granite-1 specimen in Fig. 5. The raw Raman images obtained by stitching all the 12 individual images of the observed minerals obtained from characteristic peak intensity ratios are shown in Fig. 5a. The final image (“raw image”) in Fig. 5a shows the preliminary Raman contrast map for the entire area capturing sum of intensities at 185.1 and 510.9 cm^−1^ to illustrate the location of different minerals. The definitive, unambiguous phase maps generated using our novel four-step methodology are shown in Fig. 5b. Two types of biotite were observed (biotite-1 and biotite-2), likely with the same chemical composition but different atomic structure and/or crystalline orientation due to which the characteristic Raman peaks (at 185.1, 675.7, 721.6, and 767.3 cm^−1^) have different relative intensities^41–43^. This difference was accurately recorded while obtaining contrast images and thus the minerals were mapped separately. The composite Raman phase map of the region shown in optical image (Fig. 1a) was created by color-coding the individual phase maps and combining them, resulting in the map shown in Fig. 5d. This map was obtained by overlapping all the Raman phase maps which resulted in the mineral region, a non-assigned region which corresponds to the area where none of the detected minerals were present, and an overlap region which partly includes the boundaries of minerals such as biotite and hornblende. In this region, hornblende marks the transition of the biotite to the amphibolite series^44^, which has been accurately observed due to the high resolution possible with Raman imaging.

In the elemental EDS maps shown in Fig. 5c, the gray values (from 0–black to 255–white) of each pixel are inversely proportional to the concentration of the respective element. The EDS maps thus show the presence of elements in various regions which corresponds with minerals that contain those elements. For example, the Si map shows pixels with a range of gray values which indicates varying concentration of Si across the map i.e., presence of different silicates. The Si weight concentration is the highest in quartz (0.47), followed by orthoclase (0.3), albite (0.32), hornblende (0.21–0.23) and biotite (0.17–0.21)^39^ and the same trend can be seen in the Si EDS map as well. Similarly, other elemental maps show elemental presence only in the region where the minerals which have these elements are present. As Raman spectroscopy can detect differences in mineral structure^42,46^ and crystalline orientation^41^, it was possible to distinguish biotite-1 and biotite-2 from one another, whereas with EDS such a distinction was not possible. Specifically, while the composite EDS and Raman maps show an overall good correlation in terms of the type and location of minerals, polymorphs of biotite can only be distinguished by Raman imaging. Figure 5e shows EDS and Raman composite maps of marked boxes in Fig. 5d. The area enclosed in the red and black boxes in Fig. 5d show a continuous map of quartz and a mineral boundary between albite and hornblende respectively. On one hand, a low-resolution map of EDS is good for a continuous mineral, whereas the discrepancy of EDS can be seen at mineral boundaries. In comparison, Raman maps obtained at high resolution were clearly able to map every fine detail at the mineral boundary. Table 1 has quantitative data on the level of agreement between the amount of different mineral phases observed by the two techniques. In general, there is a good level of agreement for orthoclase (99.8%), followed by albite (98.1%), hornblende (98.0%), quartz (97.3%) and biotite (93.6%). However, there is slight disagreement in the values for albite (3.6% in Raman and 6.3% in EDS map) and quartz (41.8% in Raman and 45.6% in EDS map) as a part of these minerals were overlapped in the Raman composite map. Overlap occurs at a point if the Raman spectra contains characteristic peaks from multiple minerals^24,47^. The area of the overlap region in Raman composite map exceeds that of non-assigned region in the EDS composite map for quartz and albite. This resulted in low actual mineral percentages in Raman composite map for these two minerals (41.8% and 3.6% for quartz and albite respectively). Biotite and hornblende had low overlaps only in the mineral boundaries due to which the percentages in the Raman composite map (24% for biotite and 16.2% for hornblende) did not reduce significantly. On the other hand, there are notable disagreements when it comes to the total non-assigned and overlap regions. The EDS composite map has an overall higher amount of non-assigned region, primarily due to its lower spatial resolution (5.12 μm/pixel vs 1.96 μm/pixel). EDS map has zero overlap since every point that gets scanned gets assigned to only one type of mineral based on its Si weight % value. The scan time and resolution values (84 min at 1.96 μm/pixel for Raman and 120 min at 5.12 μm/pixel for EDS) show that Raman spectroscopy is considerably better in terms of time-resolution trade-off.

**Table 1:** Mineral distribution of the selected area as quantified from composite Raman and EDS phase maps.

Sample	Mineral	Raman (%)	EDS (%)	LOA (%)	Note
Granite-1	Quartz	41.8	45.6	97.3	Lower % in Raman is due to overlap region
	Biotite	24	14.9	93.6	Lower % in EDS is due to high non-assigned region
	Hornblende	16.2	13.4	98	
	Orthoclase	5	5.3	99.8	Good level of agreement
	Albite	3.6	6.3	98.1	Lower % in Raman is due to high overlap
	Non-assigned	6.1	14.2	–	Lower resolution of EDS results in more non-assigned %
	Overlap	3.3	0	–	–
	Total	100	100	95.3	–
Granite-2	Microcline	52.5	50.9	98.9	Good level of agreement
	Albite	36.9	41	97.1	
	Quartz	2.6	1.3	99.1	
	Non-assigned	6.6	6.9	–	
	Overlap	1.4	0	–	–
	Total	100	100	97.9	–
Granite-3	Albite	95.8	98.2	98.3	Lower % in Raman is due to albite-2 being present in the cracked region
	Calcite	1.7	1.6	99.9	Good level of agreement
	Non-assigned	1.9	0.2	–	–
	Overlap	0.6	0	–	–
	Total	100	100	98.9	–
Average LOA of all 3 granites				97.4	

The fourth column shows level of agreement (LOA) for mapped phases which is defined as: 100 − σ, where σ is the standard deviation of quantitative phase percentages obtained from Raman and EDS. The average LOA (97.4%) is for the entire scan area for all 3 granites.

We adopted the developed methodology to two other granites with very different compositions (granite-2 and granite-3 abundant in microcline and albite respectively) and obtained a high level of agreement (> 97%) between the mapped phases. The corresponding phase maps are reported in supplementary Fig. 1. and the quantitative phase percentages along with the level of agreement of mapped phases observed by both the techniques are reported in Table 1. The phase percentages quantified by both techniques are compared in supplementary Fig. 2 and we report a high coefficient of determination (0.99) corroborating the developed methodology.

Finally, the back-scattered image of granite-1 in Fig. 5d shows a clear contrast between regions that contain heavy and light elements^48^, in agreement with phases observed by EDS and Raman. The brightest area corresponds to the biotite and hornblende region as it is known that these minerals contain a significant amount of Fe in them^49,50^, followed by orthoclase, albite, and quartz which have elements with relatively lower atomic weights^27^. The secondary electron image and optical image in Fig. 5d reveal the presence of surface heterogeneities and some cracking in the hornblende and quartz region, underscoring the capability of Raman imaging to work well on unpolished specimens.

## Conclusion

Our novel approach to generate quantitative mineral phase maps can, in principle, be extended to any multi-phase heterogeneous system without the need to prepare polished specimens. Several studies in the past have had limitations, such as absence of high spatial resolution maps^15,29^ as well as phase maps that are often not definitive enough^51–53^. Thus, an important implication of our results is that we can obtain high-fidelity and high-resolution phase maps of minerals without the use of thin sections or polished specimens which are often laborious to prepare. These definitive phase maps are able to quantify all present minerals, uniquely distinguish polymorphs of a mineral, and also clearly demarcate mineral transition regions. Furthermore, it was also possible to clearly differentiate minerals such as albite and orthoclase which have close principal characteristic Raman peaks by utilizing the unique peak intensity ratio method reported here. The composite phase maps of minerals generated from Raman spectroscopy and EDS had a high level of agreement proving that accurate phase mapping can be done on any rock specimen using the proposed methodology. Finally, Raman imaging has distinct advantages in quantitative mineral mapping and identification when compared to SEM–EDS such as high-spatial resolution and detection of mineral polymorphs.

## Materials and methods

### Optical imaging and Raman spectroscopy

Granite rock specimens of approximate dimension 50 × 10 × 2 mm obtained from Ward’s Science were used to perform the analysis. For granite-1 specimen, Raman Spectroscopy was performed on a scan area of 2.21 × 2.36 mm to characterize the specimen. To obtain scans in high resolution, the scan area was divided into a grid of 3 columns and 4 rows, and images (785 µm × 677 µm) were captured using a 10X objective lens (working distance—17.3 mm, numerical aperture—0.3). The stitched optical image taken in high resolution and one of the tiles used to stitch the image (tile 8 in 3rd row and 2nd column) are shown in Fig. 1. The area in Fig. 5e was captured using a 50X objective (working distance—1 mm, numerical aperture—0.8). Polarized Raman spectra were obtained (Nanophoton Raman 11) using a 532 nm laser of spot size 0.41 µm for 50X objective (1.08 µm for 10X objective) and a 600 gr/mm grating with a slit width of 50 µm. The measurement dimension in every tile using the laser was set to 785.4 × 677.4 µm and an excitation power of 0.32 mW was used. Exposure time was set to 1 sec / line of pixels totaling to 7 min per tile. The spectral resolution was 1.8 cm^−1^ and the spectra were acquired in the wavenumber range of 200 cm^−1^ to 2000 cm^−1^. The Raman spectra were subjected to baseline correction on RAMAN Viewer, proprietary software developed by Nanophoton Corporation. Raman contrast images were obtained by plotting characteristic peak intensity ratios for all the minerals at the following wavenumbers: quartz (SiO_2_) − I_466_/I_207.2_ cm^−1^ (ratio—4.5), orthoclase (KAlSiO_3_) − I_515.2_/I_508.8_ cm^−1^ (shoulders of peak at 515.2 cm^−1^, ratio—2.5), albite (NaAlSiO_3_) − I_508_/I_517_ cm^−1^ (shoulders of peak at 510.9 cm^−1^, ratio—2.5), biotite-1 (K(Mg,Fe)_3_(AlSi_3_O_10_) (OH)_2_) − I_185.1_/I_675.7_ cm^−1^ (ratio—2.7), biotite-2 (K(Mg,Fe)_3_(AlSi3O_10_)(OH)_2_) ) − (I_238_/ (I_185.1_ + I_771.4_ + I_471.0_), ratio—1.2), and hornblende ((Ca,Na)_2_(Mg,Fe,Al)_5_ (Al,Si)_8_O_22_(OH)_2_) − (I_238_/ (I_185.1_ + I_771.4_ + I_471.0_), ratio—2.5).

### Raman phase mapping

A four-step methodology was developed to obtain definitive phase maps for all minerals. Contrast images were generated after selecting characteristic peak intensity ratios for every mineral. For example, the peaks at 185.1 and 675.5 cm^−1^ were chosen for biotite. This characteristic peak intensity ratio was used to generate a contrast image whose gray value at every location is proportional to the ratio ranging from − 10 to + 10. This image was filtered for the required range of characteristic peak intensity ratios (0 to 2.7 for biotite). In this image, all the pixels that have a ratio of 0 or smaller and 2.7 or greater get assigned to a value of 0 and 255 respectively in the pixel scale. The range was so chosen such that the maximum observed ratio for a particular set of characteristic peaks was set as the upper limit. Using *ImageJ*, this image was then subjected to a Fourier transform, and subsequently a bandpass filter was applied to segment the mineral of interest from the rest. A histogram of gray values of this image was generated which had peaks corresponding to minerals present and absent. This image was then thresholded corresponding to the peaks of required minerals to generate phase maps which were essentially binary images showing presence and absence of minerals. The stepwise results for the selected tile are shown in Fig. 3. This process was adopted for minerals observed in all the tiles to obtain definitive phase maps.

### Electron imaging

Electron imaging and EDS mapping was done on unpolished, flat specimens using an environmental scanning electron microscope coupled with an Energy Dispersive X-ray Spectrometer (EDS). Secondary electron and back scattered images were captured in low vacuum mode with an accelerating voltage of 10.00 kV, spot size of 5 nm, and a chamber pressure of 1 Torr. Images were obtained with a working distance of 10 mm and 40X magnification in such a way that the entire scan area observed in the Raman microscope was visible. The EDS maps were generated with a dwell time of 250 µs and a resolution of 1024 × 800 pixels using a EDAX light element EDS module. Si, Al, Fe, Mg, K, Na, and Ca were selected based on preliminary EDS scans performed on the scan area as well as knowledge of the mineral compositions. The biotite-1 mineral (Fig. 5d) observed from Raman imaging was used as a marker to locate the same area on the SEM, using the back-scattered image. This image was used to align and orient the EDS images with the optical image obtained from the Raman spectrometer, which ensured that Raman and EDS showed the same area. The raw images obtained using both techniques were slightly larger (~ 2.5 × 2.5 mm) than the images that were adjusted for orientation, translation, and finally cropped to the same size (2.21 × 2.36 mm).

## Supplementary Information

Supplementary Information.
