# Supplementary material for: High-fidelity and high-resolution phase mapping of granites via confocal Raman imaging
Source: Sci Rep. 2021 Apr 13;11:8022. doi: 10.1038/s41598-021-87488-1 (PMC8044247; doi:10.1038/s41598-021-87488-1)
Supplement: Supplementary file 1 — Supplementary Information. [file 41598_2021_87488_MOESM1_ESM.docx]

**Supplementary Information**

**High-fidelity and high-resolution phase mapping of granites via confocal Raman imaging**

Krishna C. Polavaram,^1^ Nishant Garg^1^*

^1^Department of Civil and Environmental Engineering, University of Illinois at Urbana-Champaign, Newmark 2129, 205 N. Mathews, Urbana, IL 61801.

*Corresponding author. Email : nishantg@illinois.edu


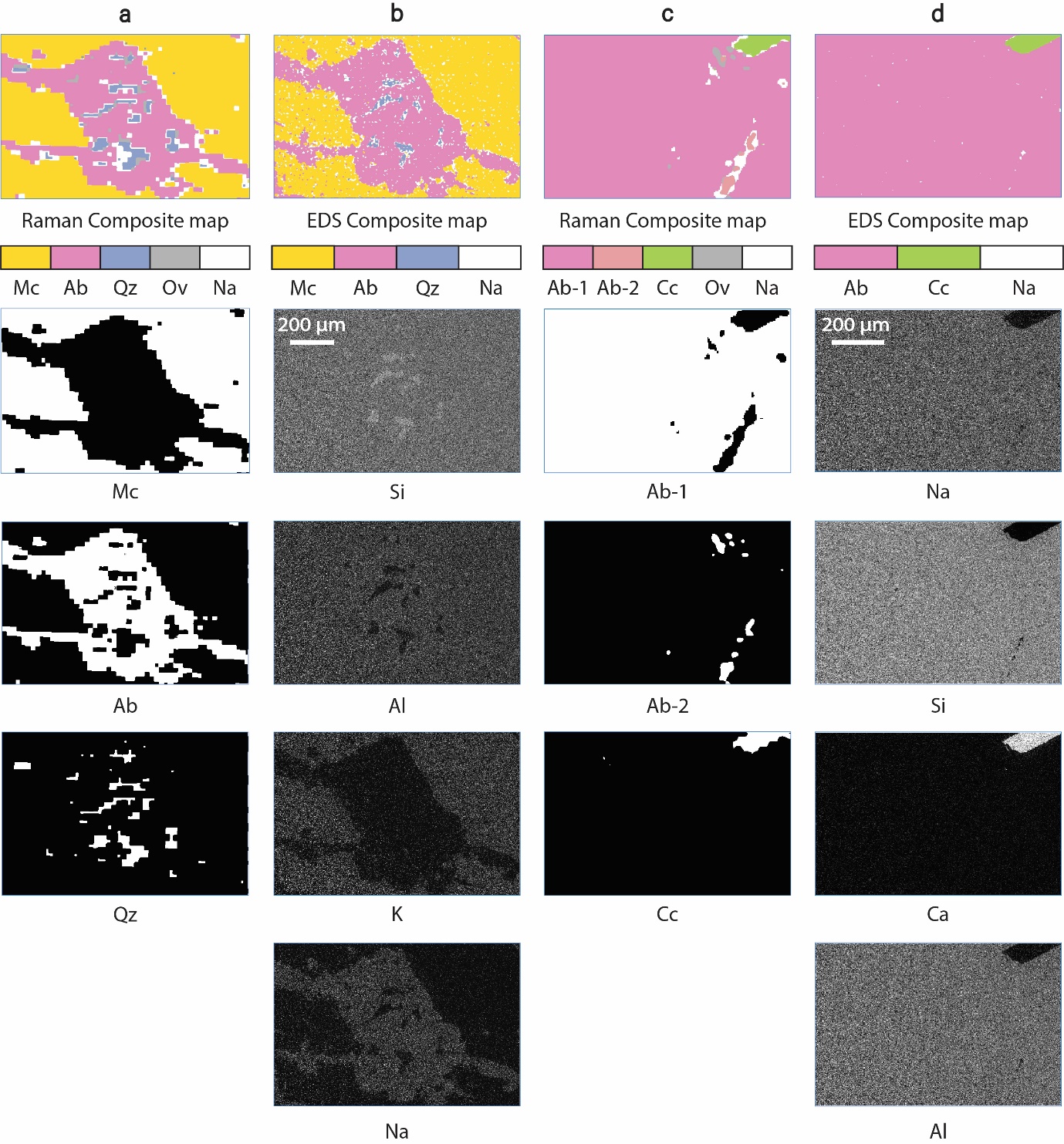


**Supplementary Figure 1:** Raman and EDS imaging on granite-2 (rich in microcline with albite and quartz as inclusions) and granite-3 (rich in albite with calcite as an inclusion) rocks using the developed methodology. (A) Raman composite and phase maps granite-2 that shows microcline, albite, quartz, an overlap, and a non-assigned region. (B) EDS composite map and elemental maps of Si, Al, K, and Na. EDS composite maps show microcline, albite, quartz, and a non-assigned region. (C) Raman composite and phase maps of granite-3 that shows albite (where albite is present as albite-1 and albite2), calcite, an overlap, and a non-assigned region. (D) EDS composite map and elemental maps of Na, Si, Ca, and Al. EDS composite map only shows albite, calcite, and a non-assigned region. Legend: Mc: microcline (KAlSi_3_O_8_), Ab: albite (NaAlSi_3_O_8_), Qz: quartz (SiO_2_), Cc: Calcite (CaCO_3_), Ov: overlapped region, Na: non-assigned. White reflects presence of mineral/ element.


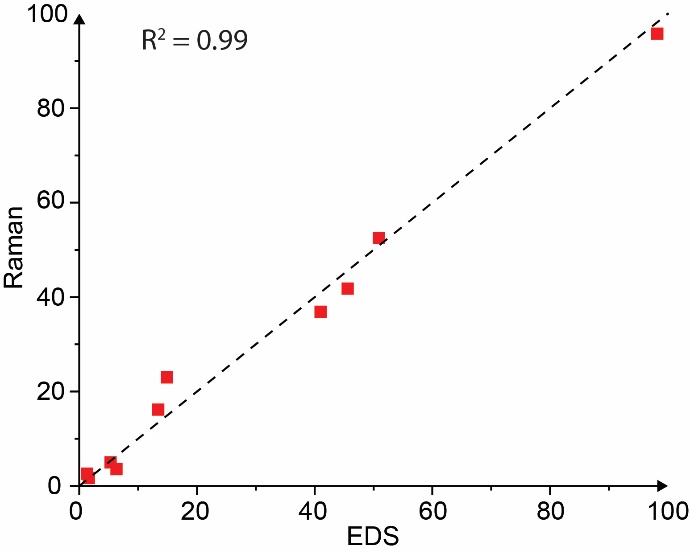


**Supplementary Figure 2:** Comparison of mineral phase percentages in all 3 granites as quantified from composite Raman and EDS maps. A high coefficient of determination of 0.99 was obtained.
